# Management of Capillary Hemangioma of the Sphenoid Sinus

**DOI:** 10.3390/medicina59050858

**Published:** 2023-04-28

**Authors:** Irina-Gabriela Ionita, Viorel Zainea, Catalina Voiosu, Cristian Dragos Stefanescu, Cristina Aura Panea, Adrian Vasile Dumitru, Ruxandra Oana Alius, Razvan Hainarosie

**Affiliations:** 1 ENT Department, Faculty of Medicine, “Carol Davila” University of Medicine and Pharmacy, 8th Eroii Sanitari Boulevard, 050474 Bucharest, Romania; 2 “Prof. Dr. D. Hociota” Institute of Phonoaudiology and Functional ENT Surgery, 21st Mihail Cioranu Street, 061344 Bucharest, Romania; 3 Neurology Department, Elias Emergency University Hospital, 17th Marasti Boulevard, 011461 Bucharest, Romania; 4 Pathology Department, Emergency University Hospital, 169th Independence Street, 050098 Bucharest, Romania

**Keywords:** capillary hemangioma sinus, endoscopic surgery, sphenoid tumor

## Abstract

*Background and objectives:* Capillary hemangiomas are rare, benign vascular tumors that mainly affect the skin and soft tissue, with scarce appearance within the nasal cavities and paranasal sinuses. *Materials and methods:* We present a case report of capillary hemangioma of the sphenoid sinus and a review of the literature in the last ten years. *Results:* Clinical and endoscopic examination of the nose, radiologic assessment and particular histologic features contribute to the correct diagnosis of capillary hemangioma of the nose and paranasal sinuses. *Conclusions:* Transnasal endoscopic resection of capillary hemangioma located in the nose and paranasal sinuses is a valuable treatment method with good outcomes.

## 1. Introduction

Capillary hemangiomas are rare, benign vascular tumors that mainly affect the skin and soft tissue. These tumors are frequently encountered in infants and children, more common in the female population, and are rarely diagnosed in adults [1].

Hemangiomas are vascular tumors that appear due to endothelial cell growth. Proliferation of regular (with normal architecture) endothelial cells is characteristic for hemangiomas. They should not be mistaken for vascular malformation, which occurs due to defects of vascular morphogenesis [2]. Vascular malformations are lesion defined by vascular anomalies determined by the alteration of embryogenesis and vasculogenesis [2].

Most hemangiomas appear on the skin in the head and neck region, but only 12% occur in the nose and paranasal sinuses with a predominance of the nasal cavity location [3,4]. Hemangiomas in the paranasal sinuses are rare findings, and most cases are reported in the maxillary sinus. Kim S.J. and Kwon S.H. reported maxillary sinus involvement in only 8% of hemangiomas affecting the sinonasal epithelium; no sphenoid sinus involvement was described in this study [4]. The same authors state that the nasal septum and inferior turbinate were the most common sites affected by hemangiomas with 40.5% and 29.7% of all cases, respectively [4,5], but other sites such as the nasal vestibule, middle turbinate, and uncinate process have been reported [6]. The presence of a capillary hemangioma within the sphenoid sinus is rare, and there are limited scientific papers regarding this specific site of appearance [1].

Intracranial hemangiomas are also rare cases, with a female predilection in adult patients, but with a possible rapid evolution, especially in pregnant women or women in the peripartum period [1,7,8]. Some authors state that pregnancy (due to all its hormonal changes) triggers the tumor’s progression and can cause recurrence of the hemangioma [8]. The location and size of the tumor will determine the clinical manifestation, but headache and visual impairment are the most frequent symptoms.

## 2. Materials and Methods

The aim of this manuscript is to present a case report of capillary hemangiomas of the left sphenoid sinus and a review of the literature in the last ten years.

We reviewed English articles published in 2013–2023 on PubMed and the Wiley Online Library. An interrogation was made on specific terms “capillary hemangioma sinus”, “capillary hemangioma nose”, and “capillary hemangioma sphenoid”. On PubMed, the search using the specific terms mentioned above revealed 35 articles, but only 14 were related to the nose and paranasal sinuses. In the Wiley Online Library, the investigation revealed 23 manuscripts with capillary hemangioma, but only 6 articles referred to the nose and paranasal sinuses. During the interrogation, we found no series of sphenoid sinus capillary hemangiomas. The articles presenting this specific localization were case reports. The included case series regarding capillary hemangioma consisted of a limited number of patients and were usually retrospective studies.

The aim of this paper is to present the case of a 31-year-old female patient referred to our clinic by a regional ENT specialist for an excruciating headache (eight out of ten on the Numeric Pain Rating Scale) experienced during a flight, which partially resolved after oral analgesic intake. She was first treated by her otolaryngologist with oral antibiotics, oral steroids, and nasal drops with improvement of the severe headache, but a computed tomography of the paranasal sinuses was recommended. There is no personal history of allergies or other medical associated conditions, no ENT- specific symptoms such as nose bleeding or rhinorrhea, and no neurologic or ophthalmologic manifestations. The patient also denied any history of head trauma or previous nasal and sinus surgery or pregnancy.

Complete physical examination and flexible optic examination of the nasal cavities, pharynx, and larynx (with white light and narrow-band imaging) revealed a nasal septum deviated to the left side, inferior turbinate hypertrophy grade III, and a pulsatile area at the level of the left sphenoethmoidal recess with an atypical vascular pattern on narrow-band imaging examination, without abnormal nasal or sinus discharge. The nasopharynx was mass-free, without signs of inflammation or secretions. On flexible endoscopy, no mass was visible within the nasal cavity or the left sphenoethmoidal recess. The rest of the specific ENT examination was normal.

**Figure 1:**
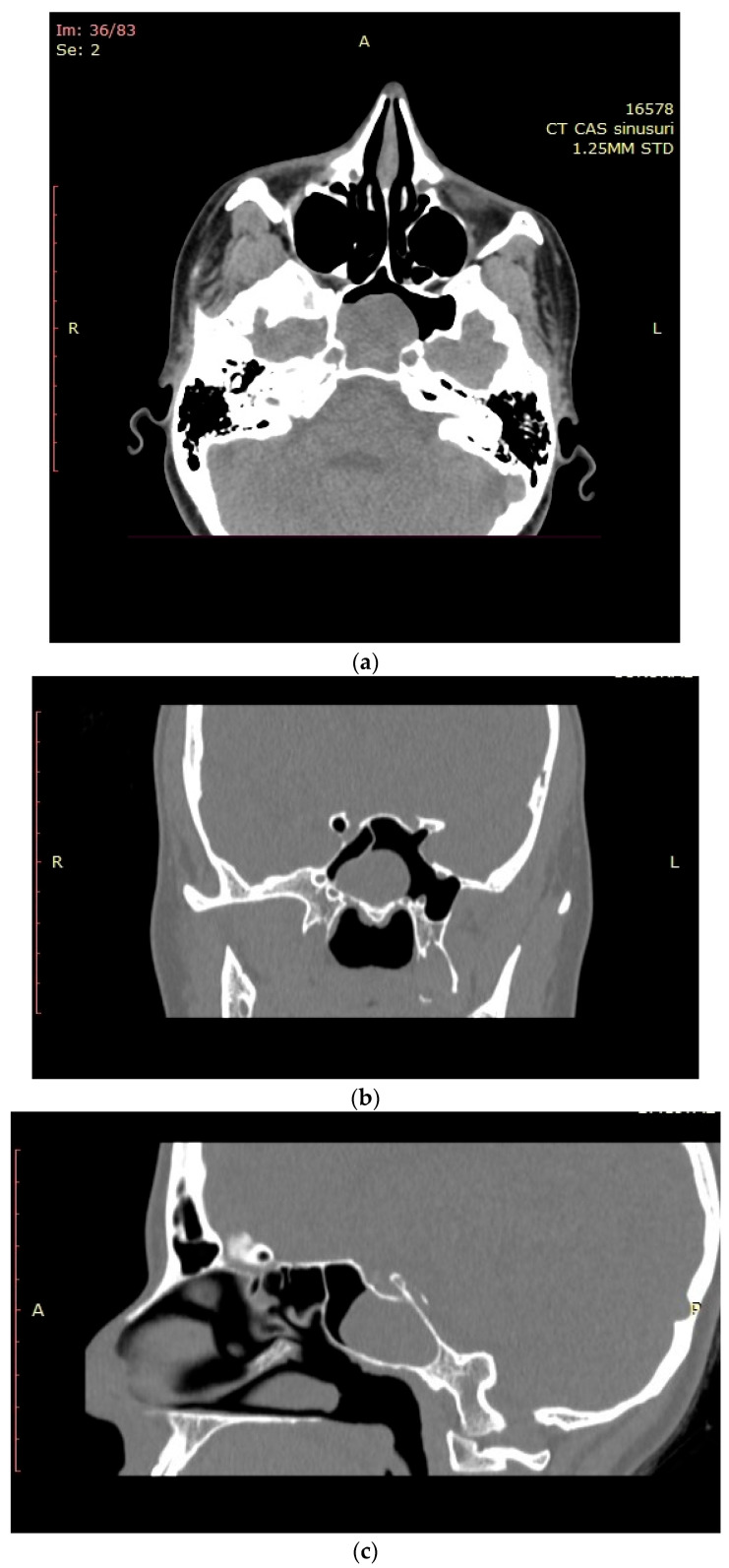
CT of the paranasal sinuses - (**a**) axial, (**b**) coronal, and (**c**) sagittal planes—lesion in the left sphenoid sinus.

The computed tomographic (CT) examination described a 32 × 28 mm pseudo-cystic lesion in the left sphenoid sinus that occupied approximately 80% of the sinus cavity, in close relation with its posterior wall. The lesion was located within the sinus cavity without extension into the sphenoethmoidal recess or the nasopharynx. No bone erosions were visible on the imaging. All other paranasal sinuses appeared well aerated and tumor-free (Figure 1a–c). The CT assessment also highlighted the nasal obstruction with left posterior nasal septum deviation that determines blockage of the left sphenoethmoidal recess and bilateral inferior turbinate hypertrophy.

**Figure 2:**
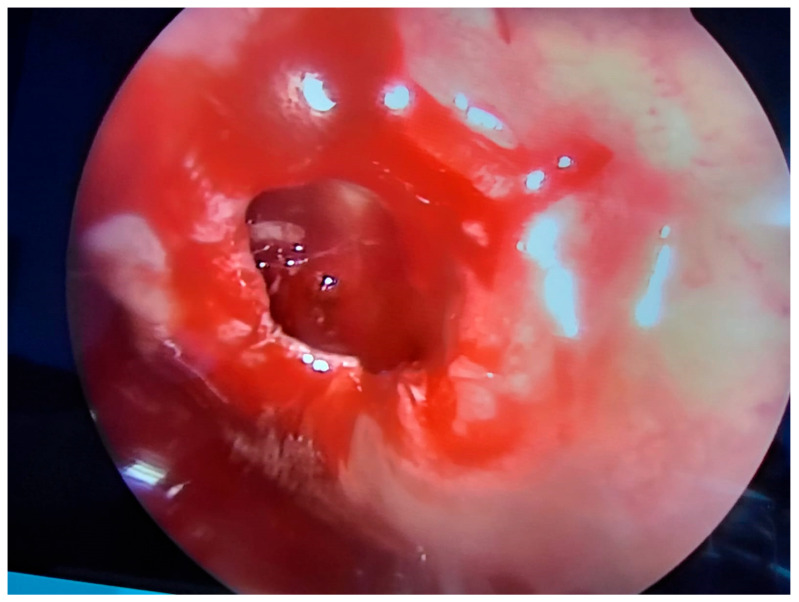
Intraoperative aspect—dissection of the mass within the left sphenoid sinus after tactical resection of the deviated nasal septum.

Upon admission to the hospital, the standard blood tests were carried out in order to establish any systemic impairment caused by the intrasinusal lesion. All blood work came back negative for any signs of inflammation or infection. Internal medicine assessment was also performed as part of the preoperative anesthesiology protocol. Written informed consent was obtained. During preoperative discussions, the goals of the surgery and the potential risks and complications, both intraoperative and postoperative, were explained to the patient. After preanesthetic assessments were checked, the patient underwent endoscopic sinus surgery to address the left sphenoid sinus under general anesthesia with orotracheal intubation. Septoplasty and inferior turbinate resection were performed to allow adequate access to the left sphenoethmoidal recess. The left sphenoid sinus was opened under endoscopic control with a zero-degree endoscope, and a reddish mass occupying approximately one-third of the sinus was identified. To adequately approach the lesion, a large opening of the sphenoid sinus was performed, and the mass was carefully detached from the posterior wall of the sphenoid sinus, ablated (Figure 2), and sent for histopathology examination. The dissection of the lesion from the wall of the left sphenoid sinus was carried out cautiously in order to avoid iatrogenic incidents. In the course of the dissection, the bleeding was limited. After removal of the tumor, the posterior wall of the left sphenoid sinus was assessed, and no bone discontinuities were observed. During surgery, we were able to appreciate the size of the intra-sphenoidal lesion as being smaller than the size measured on the computed tomography assessment. At the end of surgery, bilateral nasal packing with Merocell No. 8 was performed. The nasal packing was maintained for 48 h. Then, the nasal packing was removed with no bleeding, and nasal saline instillation was recommended at discharge. The patient did not suffer any intraoperative and postoperative complications, and the recovery was uneventful. After the surgery, all symptoms subsided, and the patient was discharged with no complaints and scheduled for follow up in one week.

**Figure 3:**
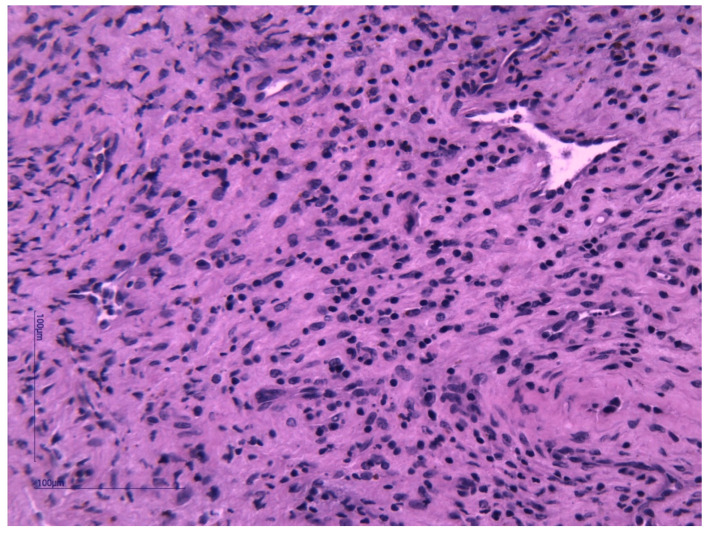
Solid area from a capillary hemangioma represented by a tumor proliferation composed of endothelial cells without atypia forming vascular structures without lumens. HE stain, ×400.

**Figure 4:**
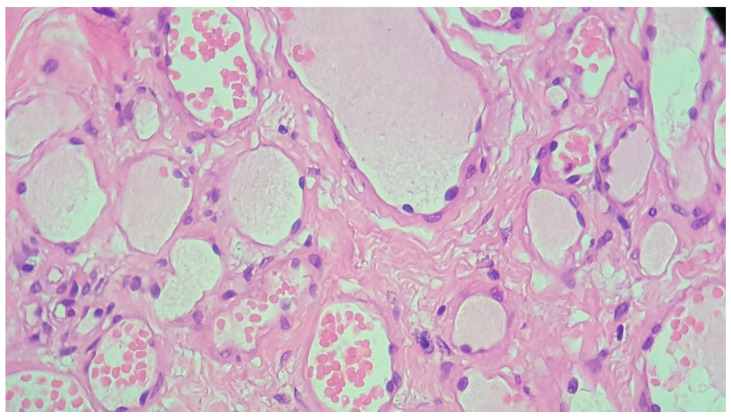
Classic appearance of a capillary hemangioma consisting of delicate vascular structures, some slightly dilated and with intraluminal red blood cells. HE stain, ×400.

Histopathological examination and immunohistochemistry established the diagnosis of capillary hemangioma with desmoplastic stroma. The pathologist described, on hematoxylin–eosin (HE) staining, tumor proliferation composed of endothelial cells without atypia, forming delicate vascular structures without lumens. The aspect was of a benign vascular proliferation formed by endothelial cells that form fragile submucosal vascular structures; some vessels appeared slightly dilated and with intraluminal red blood cells (Figure 3 and Figure 4). The pathologic assessment did not identify any ulcerations. Immunohistochemical analysis of the tumor showed that it was CD31- and CD34-positive, with Ki67 < 1%. Both immunohistochemical markers CD31 and CD34 are positive in endothelial cells, but CD31 is considered the most dependable endothelial marker [9].

**Figure 5:**
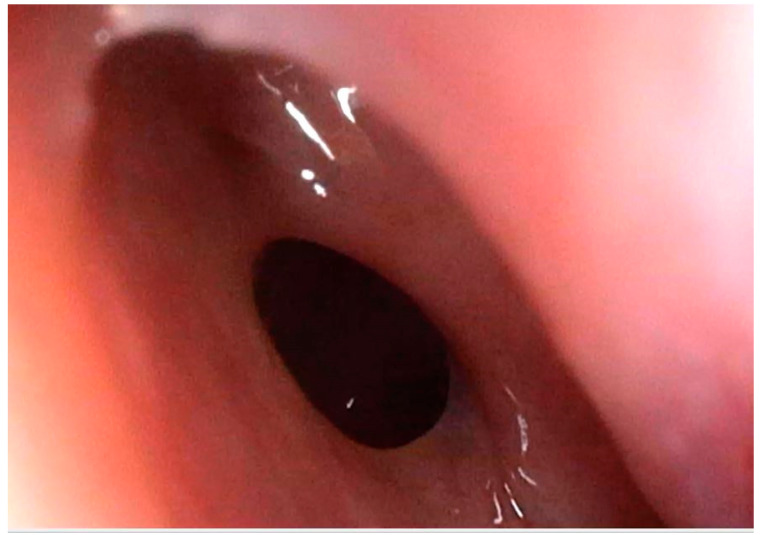
Postoperative aspect—enlarged left sphenoid sinus ostium with no signs of inflammation, no nasal or sinus discharge, and no bleeding.

**Figure 6:**
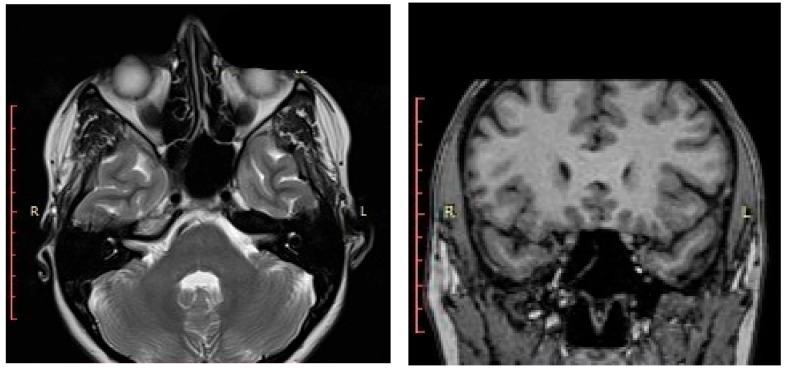
Enhanced brain MRI (axial and coronal plane)—no lesion detected within the left sphenoid sinus four months postoperatively.

After surgery, clinical and endoscopic assessments were performed every month in the first three months, afterward the visits were scheduled for every three to six months. Flexible nasal endoscopy revealed patent nasal cavities and a patent left sphenoid sinus ostium (Figure 5). No signs of bleeding or abnormal nasal discharge were visible during any of the check-ups. Moreover, the patients’ quality of life improved due to tactical removal of the deviated nasal septum and turbinoplasty. Postoperatively, the patient declared the absence of headaches and improved nasal breathing. Four months after resection, the patient received an enhanced brain (head) magnetic resonance imaging (MRI). The left sphenoid sinus was mass-free, without residual hemangioma (Figure 6).

## 3. Discussion

Hemangiomas are vascular tumors consisting of blood vessels and connective tissue that rarely affect the nose and paranasal sinuses and appear frequently on the skin in the head and neck region [9]. There are different types of classifications for hemangiomas depending on the moment of presentation and the pathologic and immunohistologic features. Hemangiomas can be congenital or infantile depending on the age of onset or presentation. From a histologic point of view, hemangiomas can be cavernous or capillary depending on the size of the blood vessels within the tumor [10]. Capillary hemangiomas have small-diameter vessels, while cavernous hemangiomas present large-diameter vessels [2,10]. Capillary hemangiomas have a characteristic pattern of vascular proliferation with a “trunk-and-branch” aspect, surrounded by pericytes, with a relative number of mitoses, fibromyoid stroma, and well-represented inflammatory infiltrate [11]. Cavernous hemangiomas have large vascular spaces lined with endothelium [12]. Ulceration of the epithelium or atrophy can appear on microscopic examination of a capillary hemangioma [13]. In the nose and paranasal sinuses, capillary hemangiomas are more frequently encountered than cavernous hemangiomas [10]. A subtype of capillary hemangioma is lobular capillary hemangioma, also known as pyogenic granuloma. It appears on the skin and in oral and nasal mucosa (especially the anterior nasal septum, turbinates, nasal vestibule, or nasopharynx) [3,4]. The etiology of lobular capillary hemangioma is not completely understood, but some theories take into account hormonal implication (particularly pregnancy and contraceptive use), injury, trauma, and viral infections [3,13,14]. Lobular capillary hemangioma can be encountered during pregnancy and is defined by the term “granuloma gravidarum”. It is located within the nasal cavities, and the main symptom is recurrent unilateral epistaxis. Managing nasal hemorrhage during pregnancy can be challenging for the ENT surgeon. The treatment should be efficient but as conservative as possible and care should be taken when prescribing certain drugs. Mohd Yusof J. et al. consider chemical cauterization with silver nitrate and anterior nasal packing to be first-line treatment for mild epistaxis during pregnancy [15]. In case of severe nose hemorrhage, surgical intervention with general anesthesia is necessary to control the epistaxis; all measures should be taken to minimize the risks of general anesthesia to the fetus [15]. Another classification of hemangiomas takes into account the presence or absence of a protein named endothelial cell glucose transporter 1 (GLUT1) [16]. In infantile hemangiomas, GLUT1 protein is expressed, whereas in congenital hemangiomas it is not [16]. A noteworthy aspect regarding hemangiomas of the nose and paranasal sinuses is their late presentation in adults (around 40 years), different from hemangiomas with other locations that appear at birth or soon afterward [10].

The symptoms can vary and depend on the location and size of the tumor, but unilateral nasal obstruction, recurrent unilateral epistaxis, and headache are among the commonest [14,17,18]. Hemangiomas are benign tumors with a slow growing rate. Usually, the patient is asymptomatic for a long period. When symptoms appear, the tumor has a considerable volume and exerts a mass effect on adjacent structures. Unilateral nasal obstruction develops progressively and advances slowly. In some cases, the nasal obstruction can be accompanied by rhinorrhea or mucopurulent nasal discharge if the drainage pathway of the affected sinus is blocked by the tumor. If the tumor is located in the anterior part of the nasal septum, unilateral recurrent anterior epistaxis is the dominant symptom. The severity of nasal hemorrhage is variable from mild to moderate and severe. In cases of mild and moderate epistaxis, local hemostatic maneuvers (nasal packing, chemical cauterization of site of bleeding) will control the bleeding. In cases of severe epistaxis, hospital admission is necessary, and emergency surgical maneuvers to control the nasal hemorrhage are imposed. A peculiarity of capillary hemangioma developing exclusively in the sinus cavity is the lack of nose bleeds in the clinical presentation of the patient. If the tumor is limited to a sinus cavity, then headache or facial pain represents the prevailing symptom. The unilaterality of the symptoms (and of the lesion) should raise suspicion of malignancy [10]. Another particular aspect of a lesion limited to the sinus cavity is the possibility of developing bony erosion through mass effect and locoregional extension without invasion into the surrounding structures. In advanced cases with orbital implication, ocular symptoms (proptosis, reduced eye mobility, impaired vision, ocular globe displacement) will accompany the nasal ones [19]. When intracranial involvement is also present, the neurologic symptoms are associated with the nasal manifestations [1]. A great range of neurologic symptoms can appear from diplopia to neurologic deficits and signs of increased intracranial pressure [8]. In the presented case, the patient had a history of chronic bilateral nasal obstruction and recurrent headaches. The intensity and particular features of the headache during the flight motivated the patient to seek medical attention. Although the symptoms were alleviated after the treatment prescribed by the general ENT specialist, the patient continued the medical investigations.

The imagistic assessment of choice is enhanced computed tomography, which allows identification of the site of the lesion, its characteristics, and its interaction with the surrounding structures. The relationship between the tumor and the base of the skull and orbit should be thoroughly checked. Binesh et al. reported two cases of ethmoid capillary hemangioma associated with bone erosion of the lamina papyracea in both adults and children; one case presented extensive bone erosion into the nasal septum and cribriform plate with extension into the anterior cranial fossa [19]. Pas M. et al. presented a case of left ethmoid and sphenoid sinus capillary hemangioma with involvement of the left cavernous sinus, extensive bone erosion, and intrasellar and parasellar extension [1].

The specific aspect of a capillary hemangioma on CT is a soft-tissue enhancing lesion associated (or not) with bone erosion located in the nose and/or paranasal sinuses [18]. According to some authors, the bone erosion is a result of the compression on the bony structures and is not an invasion of the surrounding bones [20]. Kim J.H. et al. described capillary hemangiomas from their series as well-defined, round–oval lesions, with significant enhancement at an early phase and diminished enhancement at the delayed phase on CT [12]. The same authors describe the appearance of a capillary hemangioma on MRI as low-signal masses on T1-weighted images with marked enhancement on contrast T1-weighted images and central masses surrounded by a hypo-intense peripheric rim on T2-weighted images [12].

Sometimes the histological diagnosis of capillary hemangioma can be difficult due to the endothelial proliferation pattern that resembles malignant lesions and ulceration that corresponds to granulation tissue [14]. Differential diagnosis can be made with nasal angiofibroma (when the patient is an adolescent male juvenile, angiofibroma can be taken into account), angiomatous polyp of the nose and sinuses, polypoid granulation tissue, inverted papilloma, lymphangioma, hemangiolymphangioma, glomangiopericytoma, or angiosarcoma [9,18,19,21]. Angiofibromas contain large-diameter blood vessels and stellate fibroblasts, which differ from the small-diameter vessels encountered in capillary hemangiomas [19]. Hemangiolymphangiomas are rare, lymph-containing vascular tumors characterized by multiple lymph vessels surrounded by loose fibrovascular stroma [2,21]. Glomangiopericytomas present more cells and less endothelial lining than capillary hemangiomas [19]. Angiosarcomas present malignant endothelial cells with extravascular extension and an infiltrative pattern, whilst capillary hemangiomas possess normal endothelial cells with no extravascular extension [11]. Histologic examination and immunohistochemistry establish the type of tumor. The aspect of the lesion on endoscopy and imagistic assessment can guide the surgeon, preoperatively, toward the correct diagnosis.

Surgical excision of the tumor is the preferred treatment method, and endoscopic trans-nasal surgery is most frequently used. Biopsies should be performed with care because of the high risk of bleeding due to the vascular nature of the tumor; any surgical maneuvers should be executed only after imagistic assessment [10]. Significant bleeding after biopsy should be expected when manipulating vascular tumors, and proper methods of hemostasis are imperative. Endoscopic trans-nasal removal of the tumor is considered safe and effective [22]. Furthermore, endoscopic sinus surgery is considered a minimally invasive approach for sinus hemangiomas with limited complications, reduced morbidity, and decreased hospitalization [10]. The excision can be made with electrocautery, laser, or even cryotherapy. Endoscopic surgery of intrasinusal lesions allows adequate visualization of the tumor and identification of the risk elements. Depending on the site and extension of the tumor, zero-degree or angled endoscopes can be used. Using both types (straight and angled) of endoscopes, the ENT surgeon can properly assess the sinus cavity and identify any residual tumor. Power instruments such as microdebriders should be carefully manipulated within sinus cavities, especially in the vicinity of surgical risk structures (orbit, skull base, internal carotid artery, optic nerve). Dissection with endoscopic forceps is recommended when bone erosion is identified on preoperative imagistic assessment. Preoperative CT is essential to understand the particularities of sphenoid sinus anatomy and its relation with the internal carotid artery and optic nerve—the two main risk structures in the area. Sphenoid sinus pneumatization is another factor that influences the effect on the internal carotid artery and the optic nerve. According to Fadda G.L.et al., the iatrogenic risk of injury of the aforementioned anatomic elements is higher when extensive pneumatization of the sphenoid sinus exists [23]. The presented case had significant asymmetry of the sphenoid sinus, with a small right sphenoid sinus and a wide left sphenoid sinus; this is a postsellar-type variation of pneumatization of the left sphenoid sinus. Due to the significant inequality of the sphenoid sinuses, both internal carotid arteries are in close proximity to the left sphenoid sinus. This makes them more susceptible to injury during sphenoid sinus approach. One study describes the postsellar variation as being less frequent in the studied population, but more commonly associated with protrusion of the internal carotid arteries and optic nerve within the sinus cavity [23]. Angiography with embolization may be performed before surgery, but there is no consensus among authors [6,14,20]. Preoperative embolization decreases the risk of significant bleeding during surgery and causes a regression of the tumor, allowing the ENT surgeon to manipulate the lesion and to properly dissect and ablate it. Due to the small number of reported cases and the lack of comprehensive studies, there are no available guidelines regarding the management of nasal and sinus capillary hemangiomas. Hasegawa et al. presented three cases of maxillary sinus hemangiomas that were embolized before surgery and limited perioperative bleeding was achieved (between 30 and 100 mL) [24]. Tzu-Hang C. et al. performed surgical excision of the tumor without prior embolization in all studied cases and reported no recurrence [20]. We performed endoscopic sinus surgery with no preoperative embolization in the presented case. Resection of the nasal septum and inferior turbinoplasty were necessary to create an adequate access pathway to the left sphenoid sinus and to improve nasal respiration. The particular features of the sphenoid sinus pneumatization in the presented case imposed a meticulous and prudent approach of the left sphenoid sinus cavity and its content. The choice of the adequate surgical technique and technology is adapted for every patient taking into account the effect of the technology on the healthy tissue surrounding the lesion and surgical risk elements in the vicinity [25].

The recurrence rate found in the literature varies from 0 to 42%; it depends on the study and follow-up period [20,26]. Bradshaw et al. consider that the recurrence rate depends on the technique and technology used for resection—8% recurrence rate for open excision and 40% recurrence rate for biopsy or electrocauterization—and advocate for complete resection of the tumor with a margin of healthy tissue to prevent recurrence [13]. The aforementioned authors also reported the possibility of spontaneously regression of the capillary hemangioma especially in adolescents, young adults, and pregnant women, but the regression process is not fully understood [13]. Smith et al. reported a 41.9% rate of recurrence for a mean follow-up interval of 58.6 months with a mean interval of 5.7 months between the surgery and the appearance of the recurrent tumor [26]. In their study, the presence of sinus capillary hemangiomas was limited: only one case of ethmoid lesion and no sphenoid sinus tumors. The same authors demonstrated a correlation between the age of the patient and recurrence; the mean age of the patients who developed recurrent capillary hemangioma of the nose and paranasal sinus was 50.1 years, whereas the mean age for non-recurrent tumor was 31.5 years [26]. Some authors state a 3.66% recurrence rate, but the follow-up period was relatively short (mean of 9 months) [27]. The management of recurrent capillary hemangioma is not standardized. Different approaches are available: surgical excision under general anesthesia, tumor ablation with local anesthesia, biopsy sampling, and electrocauterization. The study of Al-Ani et al., on 82 patients, demonstrated a left-side predilection of nasal capillary hemangiomas [27]. The case presented in this article was also a left-side tumor. In our case, at four months after surgery, both the endoscopic examination and brain MRI showed no residual tumor or recurrence. Taking into consideration the possibility of recurrence, we will continue follow up (clinic and endoscopic assessment every three to six months, and imagistic assessment after one year after surgery). An important aspect related to hemangioma recurrence is the lack of malignant transformation of the lesion [26].

The long-term prognosis of capillary hemangioma depends on the site of the lesion, the complete or incomplete removal of the tumor, and specific particularities of the patient (for example, pregnancy or other endocrinologic features) [8]. Given the complete removal of the tumor (assessed with enhanced head MRI four months after surgery), a favorable long-term prognosis is expected in this case. Nevertheless, the follow up will be realized as described above. The quality of life of the patient was improved after surgical resection of the tumor due to symptomatic relief. Improved nasal patency and absence of headaches were reported by the patient. The correct histologic diagnosis (histopathological examination and immunohistochemical analysis), according to the International Society for the Study of Vascular Anomalies, should be kept in mind when managing such cases [28,29].

## 4. Conclusions

Capillary hemangiomas are rare, benign vascular tumors encountered in the nose and paranasal sinuses. The clinical manifestations are not particular for this type of tumor, but the imagistic assessment can guide the surgeon to the correct diagnosis. The ENT specialist should have a high index of suspicion to diagnose capillary hemangioma within the nasal sinuses properly. Surgical resection of the tumor is the treatment of choice. Nowadays, endoscopic sinus surgery provides a good visualization of the surgical field and allows proper identification of the risk factors and correct assessment of the tumor. Complete resection of the tumor and the absence of malignant transformation (even for recurrences) are the two main features that advocate a favorable long-term prognosis for capillary hemangiomas.

Although this article represents a report of a single case of capillary hemangioma of the sphenoid sinus, the small number of cases reported in the literature make it relevant for the diagnosis and management of such tumors. Due to the lack of extensive studies of this diagnosis, there is no general consensus in its management nor in the postoperative rate of recurrence. The authors suggest that all encountered cases should undergo surveillance for a longer period of time in order to establish accurate statistical data.

## Data Availability

Not applicable.
